# Prevalence and long-term outcomes of non-alcoholic fatty liver disease among elderly individuals from the United States

**DOI:** 10.1186/s12876-019-0972-6

**Published:** 2019-04-16

**Authors:** Pegah Golabi, James Paik, Rashmi Reddy, Elisabetta Bugianesi, Gregory Trimble, Zobair M. Younossi

**Affiliations:** 1 Betty and Guy Beatty Center for Integrated Research, Inova Health System, Claude Moore Health Education and Research Building, 3300 Gallows Road, Falls Church, VA 22042 USA; 2 Department of Medical Sciences, University of Torino, Città della Salute e della Scienza, Torino, Italy; 3 Center For Liver Disease and Department of Medicine, Inova Fairfax Medical Campus, Falls Church, VA USA

**Keywords:** Epidemiology, Steatosis, Metabolic syndrome, Aging, Mortality

## Abstract

**Background:**

The prevalence and outcomes of non-alcoholic fatty liver disease (NAFLD) among elderly have not been well described. Our aim was to assess the prevalence, risk factors and mortality of NAFLD in individuals older than 60 years.

**Methods:**

The data from the Third National Health and Nutrition Examination Survey with linked mortality files were utilized. NAFLD was defined by United States Fatty Liver Index in the absence of other causes of liver disease. Cox proportional hazards models were used to assess all-cause and cardiovascular (CV) mortality. All analyses were performed using SAS software.

**Results:**

Three thousand two hundred seventy-one NHANES-III participants were included. The prevalence rates from NAFLD were 40.3% (95% CI: 37.2–43.5%) and 39.2% (95% CI: 34.4–44.0%) among 60–74 and > 74 years old. Among aged 60–74, the risks for 5-year and 10-year all-cause mortality were associated with presence of NAFLD [adjusted hazard ratios: 1.60 (95% CI: 1.24–1.96) for 5-year and 1.22 (95%CI: 1.01–1.49) for 10-year]. CV mortality were higher in this group were (aHR: 2.12 (95% CI: 1.20–3.75) for 5-year and 1.06 (95%CI: 0.73–1.52) for 10-year]. In contrast, in individuals > 74 years old, diagnosis of NAFLD was not associated with all-cause or CVD mortality.

**Conclusions:**

NAFLD is common among elderly population. Although NAFLD is associated with increased risk of mortality for 60–74-year-old individuals, this risk was not increased in those older than 74 years.

**Electronic supplementary material:**

The online version of this article (10.1186/s12876-019-0972-6) contains supplementary material, which is available to authorized users.

## Background

Non-alcoholic fatty liver disease (NAFLD) is highly prevalent in the general population worldwide [1, 2]. Most individuals with NAFLD have components of metabolic syndrome (MS) such as type 2 diabetes (T2D), dyslipidemia, hypertension and visceral obesity [3–8]. Additionally, NAFLD seems to be more common in Hispanics and less common in African Americans [9–11]. Although gender-specific differences in the prevalence of NAFLD have been debated, increasing age does seem to have an influence by increasing the prevalence of NAFLD [1, 12, 13]. However, the age cut-off chosen for the definition of “elderly” may vary across the studies, yielding conflicting results. In fact, while most studies reported an increased prevalence and severity of NAFLD in subjects older than 60 or 65 years[14], NAFLD appear to be a common but benign finding in an octogenarian population with a prevalence rate of 46.2% [15]. In fact, the prevalence of MS and co-morbidities in this group with NAFLD does not seem to be increased as compared to their counterpart without NAFLD [15]. Conversely, NAFLD patients younger than 70 years had a significantly higher prevalence of glucose intolerance, obesity and MS compared with the elderly patients with NAFLD [15]. In the Rotterdam study, the prevalence of NAFLD was lower with advancing age, ranging from 35.8% of participants < 70 years to 21.1% of participants aged ⩾85 years. Similarly, the association between number of MS criteria and probability of NAFLD weakened with advancing age [16], suggesting other mechanisms underlying the increased hepatic fat accumulation. Age-related anatomical and functional changes in the liver can also impact the progression and outcomes of liver disease [17]. In fact, older individuals lose nearly one third of their hepatic volume and perfusion, which may negatively affect the regenerative capacity of the liver [17]. In addition to the age-related lipid accumulation in non-adipose tissue, including liver, older age is also associated with sarcopenia, i.e. decreased muscle mass and function, along with dysregulation of the free radical scavenging systems which can lead to an increase in the oxidative stress potentially promoting the progression of NAFLD [18].

The higher prevalence and higher potential for progression of NAFLD, which can be associated with older age, has important implications for burden of disease. In this context, the growing burden of obesity and T2D in the United States [19] is exacerbated by the ageing of the general population [20, 21]. These two parallel trajectories have prompted important questions about the prevalence and outcomes of NAFLD in the elderly population. Therefore, the aim of this study was to assess the prevalence and long-term outcomes of NAFLD in the US population over age 60.

## Methods

This study represents an analysis of the third National Health and Nutrition Examination Survey (NHANES III) conducted by the National Center for Health Statistics (NCHS) between 1988 and 1994 [22] with the public-use linked mortality file [23]. NHANES is based on a stratified, clustered, and multistage probability sample design to obtain a representative sample of the non-institutionalized civilian U.S. population. Selected individuals participated in a home interview and also in a subsequent standardized physical examination including blood and urine collection at a mobile examination center (MEC).

Of 19,172 adults in NHANES III, 17,367 (90.6%) attend an examination at a mobile examination center. We excluded 10,784 young adults aged less than 60 years; 77 who had positive for serum hepatitis B surface antigen or hepatitis C antibody; 26 who had alcohol liver disease; 114 who had a transferrin saturation < 50%; 178 with significant alcohol consumption (≥ 20 g per day in men and ≥ 10 g per day in women). We also excluded 2917 elders with missing data on one or more components of US FLI score, leaving a study population of 3271 elders (1434 NAFLD vs. 1837 No NAFLD). Demographic characteristics and health-related behaviors, and history of comorbidities did not differ between subjects included and excluded (Additional file 1: Table S2).

General demographic characteristics collected from self-reported information included age (years), race/ethnicity (non-Hispanic white, non-Hispanic black, Mexican American, or Other race which included other Hispanic, Asian, and Native Americans), sex, and current smoker were collected from self-reported information. Serum was tested for albumin (g/dL), alanine aminotransferase (ALT, U/L), aspartate aminotransferase (AST, U/L), transferrin saturation (%), platelet count (1000 cell/uL), gamma-glutamyl transpeptidase (GGT, U/L), creatinine (mg/dL), fasting glucose (g/dL), fasting insulin (uU/mL), triglycerides (mg/dL), total cholesterol (mg/dL), high density lipoprotein (HDL, mg/dL), and low density lipoprotein (LDL, mg/dL).

### Definition of liver disease and comorbidities

For NHANES III, abdominal ultrasounds were only performed among participants ages 20–74 [24]. For the purpose of this study, the United States Fatty Liver Index (US-FLI) as a non-invasive estimate of hepatic steatosis was used to diagnose NAFLD. Recently, the US FLI have been validated in the multiethnic U.S. population with the ability to predict hepatic steatosis [25]. In this study, participants were presumed to have NAFLD, if they had a US FLI score of ≥30 in the absence of any other possible causes of chronic liver disease as described in the previous paragraphs. As a validation of US FLI, demographic and clinical parameters of adults aged 60–74 years with any degree (mild to severe) of steatosis on abdominal ultrasounds were compared with those with no hepatic steatosis (Additional file 1: Table S1). The results have great affinity with those of adults aged 60–74 years using US FLI.

*Obesity* was defined as individuals *with* BMI ≥ 30 kg/m^2^. T2D was defined as having a fasting glucose measure of > 126 mg/dL, self-reported medical history of diabetes, oral hypoglycemic, or insulin use [26]. *Hypertension (HTN)* was defined as having systolic blood pressure of > 140 mmHg or diastolic blood pressure of > 90 mmHg from an average three measurements or history of high blood pressure or history of oral antihypertensive medications. Hyperlipidemia (*HL)* was defined as a serum cholesterol level of ≥200 mg/dL, LDL of ≥130 mg/dL, and HDL ≤ 40 mg/dL in men or ≤ 50 mg/dL in women. *Metabolic Syndrome (MS)* was defined as having at least three of the following [27]: waist circumference > 102 cm in men or > 88 cm in women, fasting plasma glucose > 110 mg/dl, blood pressure > 130/85 mmHg, elevated triglycerides > 150 mg/dl, and HDL ≤ 40 mg/dL in men or ≤ 50 mg/dL in women. A homeostasis of model assessment score (HOMA) was calculated using the equation: fasting glucose (mg/dL) × fasting insulin (uU/mL)/22.7 [28]. Glomerular filtration rate (GFR) was estimated using the Chronic Kidney Disease Epidemiology Collaboration (CKD-EPI) equation for standardized creatinine [29–32]. *Chronic kidney disease (CKD)* was defined by either Urinary albumin-to-creatinine ratio (UACR) ≥ 30 mg/g or an estimated GFR of ≤60 mL/min/1.73m^2^. The *atherosclerotic cardiovascular disease (ASCVD)* risk score was calculated from each participant’s age, race, sex, smoking status, presence of diabetes, systolic blood pressure, antihypertensive medication, serum cholesterol and high density lipoprotein levels [33]. NAFLD fibrosis score (NFS) [34] and aspartate aminotransferase-to-platelet ratio index (APRI) [35], serum markers of fibrosis, were calculated according to the published formulas. *History of cardiovascular disease (CVD)* was defined by self-reported medical history of congestive heart failure, heart attack, or stroke. *History of cancer* was also defined by self-reported medical history of any cancer.

### Follow-up mortality data

The NHANES III public linked mortality file provides follow-up data on vital status from the date of NHANES III survey participation (1988–1994) through December 31, 2011 or the date of death. Mortality ascertainment is based on linking NHANES III participants with National Death Index (NDI) records through a probabilistic match [36]. Participants who were not met any death records were presumed alive through the follow-up period. Cause of death was attributed by NCHS based on the international Classification of Disease, 9th or 10th revision. Outcomes for this analysis consisted of all-cause and CVD mortality (ICD-10: I00-I09, I11, I13, I20-I25, and I60-I69) [23].

### Statistical analysis

Examination sample weights, accounting for non-response, non-coverage, and unequal selection probabilities for certain categories of the population, were incorporated in order to produce national estimates. The standard errors of estimates were estimated using Taylor series linearization, a design-based method [37]. Various socio-demographic and clinical parameters among elders (≥ 60 years old) compared by NAFLD status by using Rao-Scott chi-square for categorical variables or Wald test for continuous variables [38, 39].

To assess the effects of NAFLD on mortality outcomes, Cox proportional hazards models were utilized by adjusting demographic, metabolic syndrome, and selected comorbidities. The proportional hazards assumption of the Cox models was examined by testing time-dependent covariates [40], which showed no significant departure from proportionality over time. All analyses were performed with SAS software, version 9.4 (SAS Institute, Cary, NC) using “SURVEY” procedure, which incorporate the sample design. Statistical tests were considered significant at *p* < 0.05 (two tails).

## Results

### Baseline characteristics

Among 3271 NHANES subjects 60 years or older without any other causes of liver disease or excessive alcohol consumption (mean age 71.2 years, 42.1% male, 84.2% non-Hispanic white and 8.2% non-Hispanic black), the prevalence of NAFLD was 40.0% (95% CI: 37.5 to 42.4%). Of these, 16.0% had DM, 59.0% had HTN, 18.3% had CVD, 22.5% had MS, and 15.0% were current smokers (Table 1).

**Table 1:** Characteristics of NHANES III Participants Aged 60 and over by NAFLD status, U.S. 1988–1994

	Aged 60–74 (*n* = 2095)		Aged 75 and over (*n* = 1176)		All (*n* = 3271)
N. of NAFLD	973		461		1434
% of NAFLD (95% CI)	40.3% (37.2 to 43.5%)		39.2% (34.4 to 44.0%)		40.0% (37.5 to 42.4%)
	NAFLD	No NAFLD	NAFLD	No NAFLD	
Age, mean (SE)	67.15 (0.12)^†^	67.33 (0.17)	80.59 (0.23)^*^	81.22 (0.18)	71.16 (0.18)
Male, %	52.33 (1.74)^*†^	39.23 (1.39)	43.78 (2.06)^*^	30.82 (1.40)	42.06 (0.63)
Race, %					
non-Hispanic white	81.29 (2.17)^†^	82.82 (1.27)	89.52 (2.16)	88.80 (1.18)	84.16 (1.38)
non-Hispanic black	7.00 (0.66)^*†^	9.89 (0.89)	4.56 (0.87)^*^	8.35 (0.86)	8.20 (0.67)
Mexican American	4.35 (0.45)^*†^	1.26 (0.12)	1.89 (0.36)^*^	0.78 (0.14)	2.14 (0.18)
Other race	7.35 (1.94)	6.03 (0.98)	4.02 (1.90)	2.06 (0.53)	5.50 (1.12)
Current Smoker, %	14.85 (1.80)^*†^	19.94 (0.95)	7.24 (1.07)	8.23 (0.96)	15.02 (0.70)
Waist circumference (cm), mean (SE)	106.86 (0.30)^*†^	91.22 (0.34)	101.22 (0.55)^*^	90.65 (0.32)	96.75 (0.25)
BMI, mean (SE)	30.57 (0.19)^*†^	25.34 (0.12)	28.25 (0.22)^*^	24.67 (0.11)	27.06 (0.11)
BMI (kg/m^2^) > 30, %	46.24 (1.78)^*†^	13.24 (0.75)	31.77 (2.52)^*^	10.00 (0.86)	24.26 (0.82)
Diabetes, %	33.22 (2.18)^*^	4.06 (0.58)	32.85 (1.77)^*^	6.06 (0.76)	16.03 (0.73)
Hyperlipidemia, %	88.12 (1.19)^*^	81.38 (1.24)	86.07 (1.20)^*^	79.15 (1.51)	83.46 (0.70)
Hypertension, %	63.32 (1.63)^*†^	47.53 (1.41)	72.56 (2.02)	71.31 (1.64)	59.00 (0.86)
Metabolic Syndrome, %	44.42 (2.26)^*^	8.26 (0.62)	40.75 (2.21)^*^	9.30 (1.04)	22.49 (0.79)
History of cancer, %	17.42 (1.39)^†^	18.50 (1.52)	27.28 (1.87)	27.11 (1.38)	20.66 (0.99)
History of CVD, %	20.63 (1.28)^*†^	12.75 (1.14)	31.21 (2.35)^*^	19.86 (1.54)	18.32 (0.84)

NAFLD was defined as a US FLI score of ≥ 30 in the absence of any other cause of chronic liver disease

^*^Different from No NAFLD in the same age group (*p* < .05)

^†^Different from NAFLD Aged 75 and over (*p* < .05)

*Abbreviation*: *SE* standard error

Data displayed by weighted percentage/mean (SE)

### Baseline characteristics according to NAFLD status, stratified by age group

Among 2095 elders aged 60 to 74 years without any other possible cause of liver disease and excessive alcohol consumption, the prevalence of NAFLD was 40.3% (95% CI: 37.2 to 43.5%). Compared to non-NAFLD controls, NAFLD subjects in this age group were more likely to be male, Mexican American and more likely to have T2D, HL, HTN, MS, CVD, and had higher measurements for waist circumference, BMI, AST, ALT, ASCVD, HOMA, NFS, and APRI scores as well as lower eGFR (Tables 1 and 2). Except for fibrosis markers (APRI and NFS), which were higher in NAFLD subjects aged > 75, other characteristics of the two age subgroups were similar (Tables 1 and 2). Similar findings resulted from the validation study of using abdominal ultrasounds. The prevalence of NAFLD among adults aged 60 to 74 years was 40.7% (95% CI: 38.1 to 43.2%). However, HL and eGFR were no longer significantly different between adults aged 60 to 74 years with any degree of steatosis (mild to severe) and those with no hepatic steatosis (Additional file 1: Table S1).

**Table 2:** Clinical parameters of NHANES III Participants Aged 60 and over by NAFLD status, U.S. 1988–1994

	Aged 60–74 (*n* = 2095)		Aged 75 and over (*n* = 1176)		All (*n* = 3271)
	NAFLD	No NAFLD	NAFLD	No NAFLD	
AST (IU/L), mean (SE)	21.75 (0.27)^*^	19.98 (0.17)	21.28 (0.47)	20.51 (0.17)	20.73 (0.13)
ALT (IU/L), mean (SE)	17.83 (0.39)^*†^	12.43 (0.25)	14.26 (0.61)^*^	10.68 (0.20)	13.89 (0.29)
Albumin (g/dL), mean (SE)	4.04 (0.02)	4.02 (0.02)	3.99 (0.01)	3.98 (0.01)	4.02 (0.01)
Platelet (1000 cell/uL), mean (SE)	259.01 (3.75)	261.57 (2.90)	252.90 (2.84)	247.93 (3.39)	257.51 (2.23)
ASCVD, mean (SE)	20.80 (0.32)^*†^	14.42 (0.26)	44.39 (0.63)^*^	40.95 (0.66)	24.21 (0.42)
eGFR, mean (SE)	76.20 (0.38)^*†^	78.40 (0.48)	61.34 (0.96)^*^	65.79 (0.70)	73.67 (0.33)
HOMA, mean (SE)	7.36 (0.36)^*^	1.79 (0.03)	7.24 (0.57)^*^	1.80 (0.02)	4.01 (0.14)
NFS, mean (SE)	−0.64 (0.06)^*†^	−1.06 (0.05)	0.07 (0.06)	−0.05 (0.05)	−0.64 (0.04)
NFS, %					
> 0.676	13.92 (1.08)^*†^	6.13 (0.80)	31.54 (2.05)^*^	23.49 (1.21)	14.20 (0.84)
0.676 - -1.455	60.65 (1.86)	59.50 (1.29)	60.27 (2.14)^*^	65.78 (1.73)	61.00 (0.88)
< −1.455	25.43 (2.07)^*†^	34.38 (1.47)	8.19 (1.11)	10.74 (1.20)	24.80 (1.01)
APRI, mean (SE)	0.28 (0.01)^*^	0.25 (0.00)	0.28 (0.01)	0.27 (0.00)	0.27 (0.00)
APRI (> 0.7) %	2.05 (0.36)^*^	0.56 (0.15)	1.77 (0.76)	1.08 (0.25)	1.22 (0.14)

NAFLD was defined as a US FLI score of ≥ 30 in the absence of any other cause of chronic liver disease

^*^Different from No NAFLD in the same age group (*p* < .05)

^†^Different from NAFLD Aged 75 and over (*p* < .05)

Data displayed by weighted percentage/mean (SE)

Abbreviation: *CI* confidence interval, *ASCVD* atherosclerotic cardiovascular disease risk score, *APRI* aspartate aminotransferase to platelet ratio index, *FIB-4* fibrosis-4 score, *NFS* NAFLD fibrosis score, *eGFR* estimated glomerular filtration rate mL/min/1.73 m^2^

### Long-term outcomes of NAFLD in the elderly subjects

Median follow-up time for the study cohort aged ≥ 60 was 12.3 years (interquartile range, 6.67–18). During this period of follow up, cumulative mortality was 68.64% (2380 deaths) from all-cause and 22.71% (824 deaths) from CVD at 23 years of follow-up.

In the age cohort of 60–74, the median follow-up time was 16.5 years (interquartile range, 9.8–18.8). NAFLD subjects were more likely to die than non-NAFLD subjects (66.2% vs 54.0% from all-cause, 20.5% vs 15.7% from CVD, *p* < .01). After adjusting for age, male gender, race, smoking status, presence of metabolic syndrome, and other selected comorbidities, NAFLD had a 60% increase in the 5-year all-cause mortality (aHR = 1.60, 95% CI: 1.24–1.96), a 22% increase in the 10-year all-cause mortality (aHR = 1.22, 95% CI: 1.01–1.49), and a 23% increase in the cumulative all-cause mortality (aHR = 1.23, 95% CI: 1.07–1.42) as compared to non-NAFLD controls. Beside NAFLD, other predictors of all-cause mortality were: male sex, being smoker, hypertension, advance fibrosis (NFS > 0.676), history of CVD and cancer. Since age, race/ethnicity, and diabetes were components of US FLI and NFS scores, they were not considered in multivariable models (Table 4).

Although NAFLD was significantly associated with an increased risk of 5-year CVD mortality (aHR = 2.12, 95% CI: 1.20–3.75), it was not significantly associated with increased 10-year (aHR = 1.06, 95% CI: 0.73–1.52) and cumulative CVD mortality (aHR = 1.20, 95% CI: 0.95–1.52) (Table 3). Other predictors of CVD mortality were: being smoker, hypertension, hyperlipidemia, history of CVD and cancer (Table 4). In the group older than 75, median follow-up time was 7.5 years (interquartile range, 4.08–11.71). In this age group, presence of NAFLD had no impact on the cumulative all-cause (aHR = 1.12, 95%CI: 0.97–1.29) and CVD (aHR = 1.07, 95% CI: 0.90–1.26) mortalities (Table 3).

**Table 3:** All-cause and cardiovascular mortality among U.S. Elders, NHANES III (1988–1994)

Mortality outcome by age group	No. of subjects	No. of deaths	Cumulative mortality (%)	5-year Mortality aHR (95% CI)	10-year Mortality aHR (95% CI)	Cumulative mortality aHR (95% CI)
All-cause						
Aged 60–74						
No NAFLD	1122	643	54.02	Reference	Reference	Reference
NAFLD	973	625	66.23^*^	1.60 (1.24–1.96)^†^	1.22 (1.01–1.49)^†^	1.23 (1.07–1.42)^†^
Aged 75 +		1112				
No NAFLD	715	675	92.21	Reference	Reference	Reference
NAFLD	461	437	94.20	1.11 (0.88–1.40)	1.17 (0.99–1.36)	1.12 (0.97–1.29)
Cardiovascular disease						
Aged 60–74						
No NAFLD	917	201	15.71	Reference	Reference	Reference
NAFLD	772	188	20.46^*^	2.12 (1.20–3.75)^†^	1.06 (0.73–1.52)	1.20 (0.95–1.52)
Aged 75 +						
No NAFLD	707	260	34.96	Reference	Reference	Reference
NAFLD	458	175	36.36	1.05 (0.76–1.44)	1.13 (0.89–1.42)	1.07 (0.90–1.26)

NAFLD was defined as a US FLI score of ≥ 30 in the absence of any other cause of chronic liver disease

Multivariate models were adjusted for male, current smoker, hypertension, hyperlipidemia, advance fibrosis (NFS > .676), and history of CVD and cancer. Age, race, diabetes were not considered because of components of US FLI score

^*^Significantly different from No NAFLD (*p* < .05)

†p < .05

*abbreviation: CI* confidence interval*, aHR* adjusted Hazard Ratios

**Table 4:** Independent Predictors of all-cause and CVD morality among U.S. adults aged 60 and over, NHANES III (1988–1994)

	All-cause Mortality		CVD Mortality	
Risk Factors	Aged 60–74	Aged 75 and over	Aged 60–74	Aged 75 and over
NAFLD	1.23 (1.07–1.42)^†^	1.12 (0.97–1.29)	1.20 (0.95–1.52)	1.07 (0.90–1.26)
Male	1.41 (1.20–1.65)^†^	1.55 (1.30–1.84)^†^	1.50 (1.20–1.87)^†^	1.51 (1.21–1.87)^†^
Smoker	2.01 (1.70–2.38)^†^	1.27 (0.91–1.77)	2.09 (1.61–2.71)^†^	1.11 (0.66–1.87)
Hypertension	1.45 (1.27–1.67)^†^	1.23 (1.01–1.51)^†^	1.60 (1.26–2.03)^†^	1.24 (0.97–1.60)
Hyperlipidemia	0.96 (0.81–1.13)	1.01 (0.81–1.25)	1.35 (1.01–1.81)^†^	1.21 (0.88–1.66)
Advanced fibrosis	1.53 (1.21–1.93)^†^	1.43 (1.25–1.63)^†^	1.27 (0.88–1.82)	1.37 (1.15–1.64)^†^
History of CVD	2.00 (1.74–2.31)^†^	1.17 (0.94–1.46)	3.59 (2.86–4.49)^†^	1.59 (1.32–1.91)^†^
History of cancer	1.49 (1.24–1.78)^†^	1.15 (0.99–1.33)	1.44 (1.08–1.94)^†^	1.06 (0.90–1.25)

NAFLD was defined as a US FLI score of ≥30 in the absence of any other cause of chronic liver disease

Advance Fibrosis was defined as a NFS of ≥0.676

^†^*p* < .05

Independent predictors of all-cause mortality were older age, male sex, being smoker, diabetes, history of caner and CVD among NAFLD subjects aged 60–74, whereas older age, male, hyperlipidemia, and advanced fibrosis among NAFLD subjects aged 75 and over. On the other hand, older age, being smoker, diabetes, and history of CVD was associated with increased risk for CVD mortality among NAFLD subjects aged 60–74, whereas older age, hyperlipidemia, and history of CVD among NAFLD subjects aged 75 and over (Additional file 1: Table S3).

## Discussion

In this study, we provide data on the prevalence and the long-term outcomes of NAFLD among the elderly population in the United States. Our results indicate that NAFLD is highly prevalent in the elderly and, as expected, is closely associated with components of metabolic syndrome. In this context, there were no significant differences in the prevalence and risk factors of NAFLD among the age subgroup (60–74 vs. > 75 years old). These results are consistent with previously published data [16, 41, 42].

As described above, we have utilized US FLI as a noninvasive diagnostic method for NAFLD. For our study population, NHANES III database included ultrasound data for participants aged between 65 and 74. In this context, the prevalence of NAFLD was 40.7% by ultrasound and 40.3% by US FLI, which suggests that it is a reliable method for noninvasive diagnosis of NAFLD.

Previous data have also suggested that NAFLD may be observed more frequently in younger age in males than females [43]. In contrast, the increase in the prevalence of NAFLD in women may lag for about a decade, most likely due to estrogen changes in the post-menopausal state [18]. In fact, our results suggest that among age 60–74 years old, the majority of NAFLD patients were male. In contrast, for NAFLD subjects older than 75, the female gender was the majority.

Another interesting aspect of our data is the presence of severe fibrosis as determined by NAFLD fibrosis scores in different age groups with NAFLD. In fact, the proportion of subjects with severe fibrosis (NFS > 0.676) was higher in both NAFLD subgroups than their respective controls without NAFLD. Furthermore, the proportion of NAFLD patients with severe fibrosis (NFS > 0.676) increased as the NAFLD subjects became older (60–74 vs. > 75 years old, *p* < 0.001). This is consistent with the natural history of NAFLD suggesting the older age is a predictor of advanced fibrosis and adverse outcomes [44, 45].

The most important aspect of our study was the long-term mortality outcome of the elderly patients with NAFLD. For NAFLD group who were between 60 and 74 years old, crude cumulative mortality rates, as well as adjusted 5-year, 10-year and cumulative mortality risks were higher than the age matched controls without NAFLD. Although crude cumulative rates for CV mortality and 5-year adjusted risk for CV mortality were also high for this age group with NAFLD, 10-year and cumulative CVD mortality risks were not different than the age matched controls.

In contrast, these mortality rates for the NAFLD group older than 75 years old were not different than the age matched controls without NAFLD. These data suggest that the impact of NAFLD can only be observed up to a certain age (age 75) after which it is diluted by other age-related causes of mortality.

To our knowledge, there have been no longitudinal studies that have examined the epidemiologic and outcomes assessment of NAFLD in the elderly. The strength of this study was using population-based data from NHANES. Although US FLI has been extensively validated for diagnosing NAFLD, we are not able to provide more detailed histologic data about the subtypes of NAFLD in the elderly. Although this type of data would be valuable, obtaining histologic and outcomes data from a large population-based cohort is not feasible. Moreover, in order to detect advanced fibrosis among patients with NAFLD, we utilized NAFLD Fibrosis Score, which is a validating scoring system for prediction of fibrosis. This scoring system includes age as a variable, which might cause a bias when we emphasize the more advanced fibrosis among elderly, given their advanced age.

In summary, this in-depth assessment of NAFLD suggests a very high prevalence of NAFLD in the elderly population in the United States. Although NAFLD is associated with increased risk of mortality in those between 60 and 74, the risk is not increased in subject older than 75. These data should inform clinician and policy makers to better understand the burden of NAFLD in the ageing population of the United States [2, 44–48].

## Conclusions

Non-alcoholic fatty liver disease (NAFLD) is a growing global health problem, affecting almost a quarter of the world’s population. NAFLD is highly prevalent in the elderly and is closely associated with components of metabolic syndrome. Although NAFLD is associated with increased risk of mortality for 60–74-year-old individuals, this risk was not increased in those older than 74 years.

## Additional file

Additional file 1: **Table S1.** Characteristics of NHANES III Participants Aged 60 to 74 year, by NAFLD status, U.S. 1988–1994. **Table S2.** Characteristics of NHANES III Participants Aged 60 and over, by Inclusion Criteria of the study, U.S. 1988–1994. **Table S3a.** Independent Predictors of all-cause mortality among NAFLD elders, NHANES III (1988–1994). **Table S3b.** Independent Predictors of CVD mortality among NAFLD elders, NHANES III (1988–1994). (DOCX 22 kb)

## Abbreviations

APRI: Aspartate aminotransferase-to-platelet ratio index
ASCVD: Atherosclerotic cardiovascular disease
CVD: Cardiovascular disease
HL: Hyperlipidemia
HTN: Hypertension
MS: Metabolic syndrome
NAFLD: Non-alcoholic fatty liver disease
NDI: National Death Index
NFS: NAFLD fibrosis score
NHANES III: National Health and Nutrition Examination Survey
T2D: Type 2 diabetes
US-FLI: United States Fatty Liver Index
