# Supplementary material for: Prevalence and long-term outcomes of non-alcoholic fatty liver disease among elderly individuals from the United States
Source: BMC Gastroenterol. 2019 Apr 16;19:56. doi: 10.1186/s12876-019-0972-6 (PMC6469055; doi:10.1186/s12876-019-0972-6)
Supplement: Supplementary file 1 — Table S1. Characteristics of NHANES III Participants Aged 60 to 74 year, by NAFLD status, U.S. 1988–1994. Table S2. Characteristics of NHANES III Participants Aged 60 and over, by Inclusion Criteria of the study, U.S. 1988–1994. Table S3a. Independent Predictors of all-cause mortality among NAFLD elders, NHANES III (1988–1994). Table S3b. Independent Predictors of CVD mortality among NAFLD elders, NHANES III (1988–1994). (DOCX 22 kb) [file 12876_2019_972_MOESM1_ESM.docx]

| **Additional file 1: Table S1**. Characteristics of NHANES III Participants Aged 60 to 74 year, by NAFLD status, U.S. 1988 - 1994 | | | | |
| --- | --- | --- | --- | --- |
|  | By US FLI | | By Ultrasound | |
| % of NAFLD (95% CI) | 40.3% (37.2% to 43.5%) | | 40.69% (38.1% to 43.3%) | |
|  | NAFLD | No NAFLD | NAFLD^†^ | No NAFLD^†^ |
| Age, mean (SE) | 67.15 (0.12) | 67.33 (0.17) | 67.15 (0.21) | 67.16 (0.16) |
| Male, % | 52.33 (1.74)^*^ | 39.23 (1.39) | 48.40 (2.34)^*^ | 41.28 (1.43) |
| Race, % |  |  |  |  |
| non-Hispanic white | 81.29 (2.17) | 82.82 (1.27) | 83.91 (1.93) | 82.23 (1.62) |
| non-Hispanic black | 7.00 (0.66)^*^ | 9.89 (0.89) | 7.38 (0.68)^*^ | 9.33 (0.91) |
| Mexican American | 4.35 (0.45)^*^ | 1.26 (0.12) | 3.44 (0.33)^*^ | 2.31 (0.24) |
| Current Smoker, % | 14.85 (1.80)^*^ | 19.94 (0.95) | 15.39 (1.39)^*^ | 21.02 (1.46) |
| Waist (cm), mean (SE) | 106.86 (0.30)^*^ | 91.22 (0.34) | 102.87 (0.59)^*^ | 93.27 (0.39) |
| BMI, mean (SE) | 30.57 (0.19)^*^ | 25.34 (0.12) | 29.41 (0.24)^*^ | 25.78 (0.13) |
| BMI (kg/m2)> 30, % | 46.24 (1.78)^*^ | 13.24 (0.75) | 40.21 (2.12)^*^ | 15.32 (1.01) |
| Diabetes , % | 33.22 (2.18)^*^ | 4.06 (0.58) | 24.97 (1.84)^*^ | 9.52 (1.01) |
| Hyperlipidemia, % | 88.12 (1.19)^*^ | 81.38 (1.24) | 85.45 (1.91) | 83.73 (1.65) |
| Hypertension, % | 63.32 (1.63)^*^ | 47.53 (1.41) | 59.85 (2.73)^*^ | 48.43 (1.23) |
| Metabolic Syndrome, % | 44.42 (2.26)^*^ | 8.26 (0.62) | 35.10 (2.03)^*^ | 12.90 (0.97) |
| History of cancer, % | 17.42 (1.39) | 18.50 (1.52) | 17.18 (1.39) | 17.60 (1.30) |
| History of CVD, % | 20.63 (1.28)^*^ | 12.75 (1.14) | 18.83 (1.80)^*^ | 13.49 (1.41) |
| AST (IU/L), mean (SE) | 21.75 (0.27)^*^ | 19.98 (0.17) | 21.52 (0.30)^*^ | 20.34 (0.24) |
| ALT (IU/L), mean (SE) | 17.83 (0.39)^*^ | 12.43 (0.25) | 16.68 (0.37)^*^ | 13.39 (0.26) |
| Albumin (g/dL), mean (SE) | 4.04 (0.02) | 4.02 (0.02) | 4.08 (0.02)^*^ | 4.04 (0.02) |
| Platelet (1,000 cell/uL), mean (SE) | 259.01 (3.75) | 261.57 (2.90) | 262.07 (4.41) | 266.92 (3.19) |
| ASCVD, mean (SE) | 20.80 (0.32)^*^ | 14.42 (0.26) | 19.00 (0.35)^*^ | 15.48 (0.31) |
| eGFR, mean (SE) | 76.20 (0.38)^*^ | 78.40 (0.48) | 77.00 (0.71) | 78.11 (0.40) |
| HOMA, mean (SE) | 7.36 (0.36)^*^ | 1.79 (0.03) | 5.61 (0.35)^*^ | 2.81 (0.19) |
| NFS, mean (SE) | -0.64 (0.06)^*^ | -1.06 (0.05) | -0.80 (0.07)^*^ | -1.09 (0.05) |
| APRI, mean (SE) | 0.28 (0.01)^*^ | 0.25 (0.00) | 0.27 (0.01)^*^ | 0.25 (0.01) |
| *^†^ NAFLD was defined as any degree of hepatic steatosis on abdominal ultrasounds in the absence of any other cause of chronic liver disease. ^*^ Different from No NAFLD (p<.05) Abbreviation: SE, standard error Data displayed by weighted percentage/mean (SE)* | | | | |

| **Additional file 1: Table S2.** Characteristics of NHANES III Participants Aged 60 and over, by Inclusion Criteria of the study, U.S. 1988 - 1994 | | | |
| --- | --- | --- | --- |
|  | Elders included | Elders excluded^†^ | P |
| **N** | 3,271 | 2,917 |  |
| **Age, mean (SE)** | 71.16 (0.18) | 71.52 (0.30) | 0.3888 |
| **Male, %** | 42.06 (0.96) | 42.34 (1.51) | 0.8828 |
| **Race, %** |  |  |  |
| non-Hispanic white | 84.16 (1.92) | 84.08 (2.62) | 0.9820 |
| non-Hispanic black | 8.20 (1.03) | 9.54 (1.61) | 0.5293 |
| Mexican American | 2.14 (0.31) | 2.81 (0.78) | 0.4797 |
| Other race | 5.50 (1.23) | 3.57 (1.08) | 0.2470 |
| **Current Smoker, %** | 15.02 (1.08) | 18.08 (1.72) | 0.1556 |
| **Waist (cm), mean (SE)** | 96.75 (0.25) | 96.41 (0.39) | 0.4702 |
| **BMI, mean (SE)** | 27.06 (0.11) | 26.71 (0.19) | 0.1359 |
| **BMI (kg/m^2^)> 30, %** | 24.26 (0.98) | 22.75 (1.39) | 0.3090 |
| **Diabetes , %** | 16.03 (0.93) | 18.53 (1.69) | 0.1711 |
| **History of cancer, %** | 20.66 (1.33) | 19.45 (1.59) | 0.5589 |
| **History of CVD, %** | 18.32 (1.16) | 20.64 (1.73) | 0.1914 |
| ^†^elders with missing data on one or more components of US FLI score Data displayed by weighted percentage/mean (SE) | | | |

| **Additional file 1:** Table S3a. Independent Predictors of all-cause mortality among NAFLD elders, NHANES III (1988-1994) | | | | |
| --- | --- | --- | --- | --- |
| **Covariate** | Aged 60 - 74 | | Aged 75 and over | |
|  | HR (95% CI) | P | HR (95% CI) | P |
| **Age** | 1.08 (1.05 - 1.11) | <.0001 | 1.09 (1.07 - 1.12) | <.0001 |
| **Male** | 1.20 (1.02 - 1.42) | 0.0267 | 1.46 (1.15 - 1.86) | 0.0029 |
| **Race, %** |  | 0.6731 |  | 0.0400 |
| non-Hispanic white | Reference |  | Reference |  |
| non-Hispanic black | 1.08 (0.85 - 1.37) | 0.5381 | 0.84 (0.57 - 1.23) | 0.3545 |
| Mexican American | 0.96 (0.83 - 1.10) | 0.5077 | 0.78 (0.54 - 1.13) | 0.1812 |
| Other race | 1.13 (0.80 - 1.60) | 0.4831 | 0.31 (0.13 - 0.73) | 0.0084 |
| **Current Smoker** | 1.90 (1.54 - 2.34) | <.0001 | 1.16 (0.80 - 1.68) | 0.4273 |
| **Diabetes** | 1.57 (1.25 - 1.97) | 0.0003 | 1.08 (0.85 - 1.37) | 0.5405 |
| **Hypertension** | 1.26 (0.98 - 1.62) | 0.0730 | 1.06 (0.86 - 1.30) | 0.5866 |
| **Hyperlipidemia** | 1.02 (0.70 - 1.47) | 0.9193 | 1.23 (1.03 - 1.46) | 0.0231 |
| **Advanced fibrosis** | 1.15 (0.83 - 1.61) | 0.3894 | 1.25 (1.00 - 1.55) | 0.0469 |
| **History of Cancer** | 1.41 (1.11 - 1.80) | 0.0062 | 1.23 (1.00 - 1.50) | 0.0516 |
| **History of CVD** | 1.87 (1.50 - 2.33) | <.0001 | 1.27 (1.06 - 1.54) | 0.0133 |

| **Additional file 1:** Table S3b. Independent Predictors of CVD mortality among NAFLD elders, NHANES III (1988-1994) | | | | |
| --- | --- | --- | --- | --- |
| **Covariate** | Aged 60 - 74 | | Aged 75 and over | |
|  | HR (95% CI) | P | HR (95% CI) | P |
| **Age** | 1.07 (1.01 - 1.13) | 0.0189 | 1.11 (1.08 - 1.15) | <.0001 |
| **Male** | 1.40 (0.92 - 2.13) | 0.1162 | 1.35 (0.98 - 1.87) | 0.0684 |
| **Race, %** |  | 0.2979 |  | 0.2199 |
| non-Hispanic white | Reference |  | Reference |  |
| non-Hispanic black | 0.73 (0.39 - 1.35) | 0.3039 | 0.90 (0.46 - 1.74) | 0.7422 |
| Mexican American | 1.19 (0.84 - 1.69) | 0.3200 | 0.78 (0.52 - 1.18) | 0.2317 |
| Other race | 0.51 (0.18 - 1.48) | 0.2074 | 0.39 (0.15 - 1.02) | 0.0541 |
| **Current Smoker** | 2.61 (1.71 - 3.98) | <.0001 | 0.96 (0.49 - 1.87) | 0.8950 |
| **Diabetes** | 2.13 (1.57 - 2.89) | <.0001 | 1.08 (0.73 - 1.61) | 0.6854 |
| **Hypertension** | 1.29 (0.82 - 2.02) | 0.2676 | 0.77 (0.56 - 1.05) | 0.0917 |
| **Hyperlipidemia** | 1.59 (0.65 - 3.88) | 0.3011 | 2.44 (1.65 - 3.61) | <.0001 |
| **Advanced fibrosis** | 0.74 (0.40 - 1.39) | 0.3403 | 0.90 (0.64 - 1.26) | 0.5168 |
| **History of Cancer** | 1.42 (0.92 - 2.20) | 0.1099 | 1.25 (0.94 - 1.68) | 0.1239 |
| **History of CVD** | 2.88 (1.99 - 4.15) | <.0001 | 1.59 (1.17 - 2.17) | 0.0046 |
